# Non-invasive evaluation of labial gingival and alveolar crest thickness in the maxillary anterior teeth region by 15-MHz B-mode ultrasonography

**DOI:** 10.1186/s12903-020-01377-z

**Published:** 2021-01-06

**Authors:** Meng Sun, Xiaofeng Liu, Ting Xia, He Meng

**Affiliations:** 1 Department of Stomatology, Shenzhen University General Hospital and Shenzhen University Clinical Medical Academy, Liuxian Street, Nanshan district, Shenzhen, 518055 Guangdong China; 2 Department of Ultrasound Diagnostics and Treatment, Shenzhen Second People’s Hospital, First Affiliated Hospital of Shenzhen University, Shenzhen, Guangdong China

**Keywords:** Gingiva, Alveolar, Periodontal, Ultrasonography

## Abstract

**Background:**

Knowledge of gingival thickness (GT) and alveolar crest thickness (ACT) is essential when performing surgical and non-surgical procedures in the maxillary anterior teeth region. This study aimed at evaluating the GT and ACT in the maxillary anterior teeth region using 15-MHz B-mode Ultrasonic (US).

**Methods:**

A total of 300 teeth from 50 healthy participants, comprising 25 women and 25 men, aged between 18 and 35 years were analyzed. We measured labial periodontal tissue structures of maxillary anterior teeth, including GT and ACT, at 3 mm apical to the gingival margin (GT3) and the crestal level, respectively. The GT and ACT measurements were correlated.

**Results:**

The mean labial GT3 of the maxillary central incisors, lateral incisors, and canines were 1.24 ± 0.03 mm, 1.21 ± 0.03 mm and 1.11 ± 0.03 mm, respectively. Canine GT3 was significantly thin than those in the central and lateral incisors (*P* < 0.05). With regards to labial ACT, we recorded 0.79 ± 0.03 mm, 0.76 ± 0.02 mm and 0.73 ± 0.02 mm for maxillary central incisors, lateral incisors and canines, respectively. There were no significant differences in ACT of maxillary anterior teeth (*P* > 0.05). GT3 of men was greater than that of women (*P* < 0.05). In addition, GT and ACT were positively correlated (*r* = 0.32, *P* < 0.01).

**Conclusion:**

15-MHz B-mode US is an effective tool for measuring labial GT and ACT of anterior teeth. There are sex-associated differences in GT3 and the correlation between the GT3 and ACT of anterior teeth is moderately positive.

## Background

In clinical practice, the proper diagnosis of labial gingiva thickness (GT) and alveolar crest thickness (ACT) of the anterior teeth is important to inform decisions regarding esthetic implant dentistry, periodontal therapies, and orthodontics [1–3]. Studies have documented that a good implant esthetic outcome is easily obtained with a thick rather than a thin soft and hard tissue [4, 5]. Therefore, awareness of the initial GT and ACT is vital for treatment and prognosis.

Different approaches have been used to assess gingival biotypes and the underlying alveolar bone. The GT and ACT measuring approaches can be divided into invasive and non-invasive groups. Invasive approaches include the application of periodontal probes or needles to directly measure gingival or alveolar bone thickness after local anesthesia [6–9]. Non-invasive GT measuring approaches involve visual assessment of the general appearance of the gingiva or transparency of the periodontal probe through the gingival margin [10, 11]. However, the assessment of GT by tissue inspection is unreliable [12, 13]. Consequently, Cone-beam computed tomography (CBCT) is a non-invasive method used to visualize an individual tooth or dentition in relation to surrounding skeletal tissues and to create three-dimensional images of the area to be examined [14–16]. A common critique of CBCT is that it cannot accurately measure soft tissue. When compared with a medical CT scan, soft tissue contrast on CBCT is poor because of lower signal-to-noise ratio caused by scattering radiation from larger projection image dimensions and lower tube current [17]. Fourie et al. found that CBCT adequately measured soft tissue thickness in the facial region but recommended a 0.3 mm voxel size for enhanced accuracy [18]. Moreover, radiation exposure and the associated cost-benefits of CBCT imaging limit its effective use.

Ultrasound (US) is a non-invasive and non-radiation loaded diagnostic tool widely used in medical imaging and dentistry [19, 20]. A-mode US has been used to measure GT in cadavers or humans [21–24]. Even though the A-mode US exhibits a degree of efficiency in GT measurement, it cannot provide an image of periodontal structures or analyze their relationships. High-frequency B-mode US can be used to measure the GT and ACT. Furthermore, it can also be used to detect periodontal tissue structures with dynamic image analysis. A recent study using cadavers revealed that 14 MHz B-mode US is as accurate as CBCT when determining the alveolar bone level and thickness [25]. In addition to measuring cadaver GT, Zimbran et al. [26] used a very high frequency (40 MHz) B-mode US to measure GT of bicuspids in the mandibular region. They found that B-mode US is a highly accurate and non-invasive technique for measuring GT. However, very high frequency B-mode US is not commonly used in clinical practice, and its popularity is limited. Therefore, we aimed at evaluating the labial GT and ACT of anterior human teeth using B mode US and a 15-MHz probe.

## Methods

### Sample selection

This study was approved by the Research Ethics Committee of the Shenzhen University General Hospital (SUGHME-06004). Study participants comprised of students, staff of Shenzhen University, and patients of the General Hospital’s Department of Stomatology for non-periodontal diseases. All study participants provided a written informed consent for clinical and US examination. Clinical examination involved measuring pocket depth and bleeding using a periodontal probe (Hu-Friedy, IL, Chicago, USA). Periodontally healthy adults (pocket depth ≤ 3 mm with no bleeding or attachment loss) were enrolled in the study. Participants with ≥ 1 of the following conditions were excluded: systemic diseases with oral manifestations; drug-associated changes in periodontal tissues; presence of endodontic pathology in the anterior teeth region; previous periodontal surgical procedures; mal-positioning, crowding, abnormal teeth morphology or spacing; history of orthodontic treatment; skeletal or maxillofacial abnormalities; pregnancy or lactation; oral breathing and smoking.

### Clinical B-mode US examination

Fifty periodontally healthy participants comprising 25 males and 25 females were enrolled in the study. Their ages ranged from 18 to 35 years, with a mean of 25.8 ± 4.4 years. A total of 300 teeth were analyzed as follows; the anterior teeth were imaged using a 15-MHz transducer (Logiq E9, General Electric Company, Boston, MA, USA), and a probe (ML6-15) advanced from the labial side. The probe was coated with a coupling agent (Yizhi TM, Yijie, Guangzhou, China) on the labial side of the upper lip, and its frequency adjusted to 15-MHz. Image depth and gain were adjusted. The focus was adjusted to the gingival depth level. The probe was scanned on the maxillary transverse section on the lip surface to identify the maxillary anterior teeth, placed at the center of the tooth and rotated 90°. In the sagittal plane, the labial thickness of the soft and hard tissues of the teeth and periodontal tissues, including GT and ACT, was measured. Measurement was carefully done to prevent the US coupling agent from aspirating into the nostrils through the small maxillary space. All measurements were done in triplicates by an experienced sonographer.

### Statistical analysis

Data were analyzed using SPSS 20.0 software (IBM Corp., Armonk, NY, USA). The GT3 and ACT of anterior teeth were calculated and presented as mean ± SD. Variations in the GT3 and ACT of anterior teeth were compared using One-way analysis of variance (ANOVA) followed by least-significant difference (LSD) post hoc test and variations in the GT3 and ACT between male and female were compared using Student’s *t*-test. Further comparisons were performed using the Spearman’s correlation coefficient. *P* ≤ 0.05 was considered to be statistically significant.

## Results

**Fig. 1:**
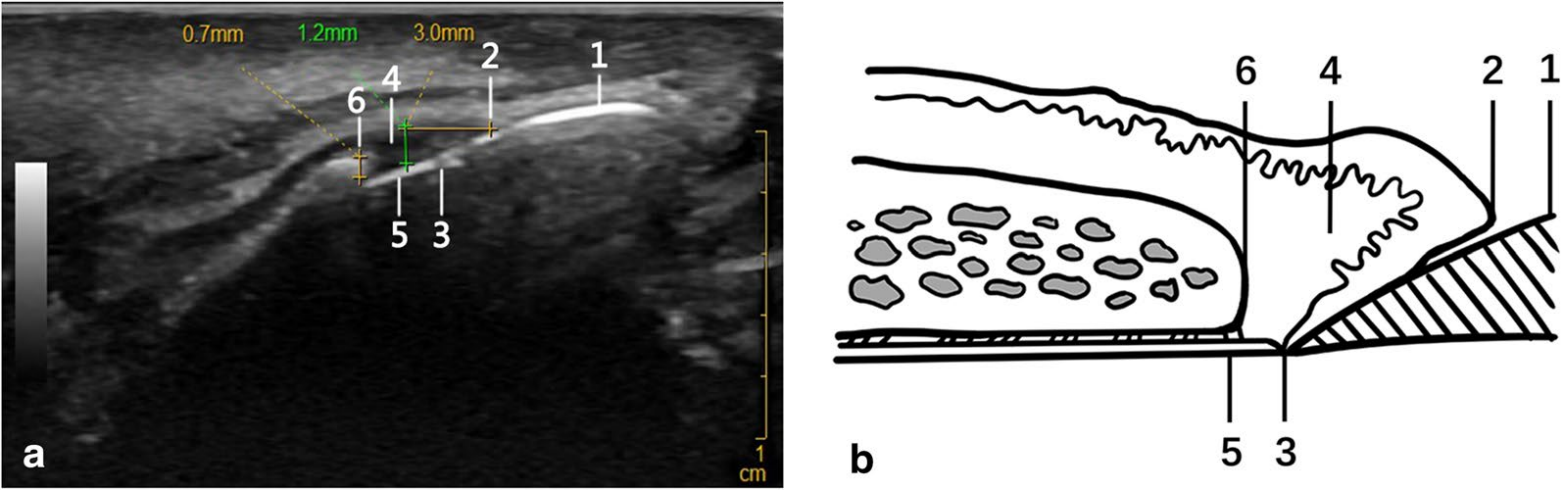
A 15-MHz B-mode ultrasonographic image of periodontal structures of a maxillary incisor and anatomic schema. **a** A sagittal ultrasonographic image through a maxillary incisor. GT3 was measured at the 3 mm level apical to gingival margin while ACT was measured at the alveolar crest. **b** Schematic of morphometrical analysis of periodontal tissues in a US image. Tissues are indicated as follows; 1, enamel; 2, gingival margin; 3, cementoenamel junction; 4, gingiva; 5, cementum and 6, the crest of alveolar bone

We used 15-MHz B-mode US to measure the GT3 and ACT of 300 teeth and obtained high-resolution grayscale images at the desired levels (Fig. 1). The images show the periodontal tissue structures. The mean labial GT3 of the maxillary anterior teeth ranged between 1.31 ± 0.04 mm and 1.05 ± 0.04 mm, while labial ACT ranged between 0.82 ± 0.04 mm and 0.71 ± 0.03 mm (Table 1). Comparisons of male and female GT3 revealed a thinner gingiva among females. Notably, canine GT3 was thinner than central and lateral incisor GT3s (*P* < 0.05). In addition, there was a moderate positive correlation between GT3 and ACT (*r* = 0.32, *P* < 0.01).

**Table 1:** Labial thickness of GT3 and ACT in maxillary anterior teeth

	Central incisor		Lateral incisor		Canine	
	Number	Mean ± SD	Number	Mean ± SD	Number	Mean ± SD
GT3	100	1.24 ± 0.03a	100	1.21 ± 0.03b	100	1.11 ± 0.03 a,b
Male	50	1.31 ± 0.04*	50	1.27 ± 0.04#	50	1.16 ± 0.04△
Female	50	1.18 ± 0.04*	50	1.16 ± 0.04#	50	1.05 ± 0.04△
ACT	100	0.79 ± 0.03	100	0.76 ± 0.02	100	0.73 ± 0.02
Male	50	0.82 ± 0.04	50	0.80 ± 0.03	50	0.76 ± 0.03
Female	50	0.76 ± 0.04	50	0.73 ± 0.03	50	0.71 ± 0.03

There were significant differences by gender at all measuring points in maxillary GT3. The GT3 of canine was thinner than that of central and lateral incisor, the difference was statistically significant, *, #, △ *P* < 0.05, a *P* < 0.05, central incisor compared to canine, b *P* < 0.05, lateral incisor compared to canine

**Fig. 2:**
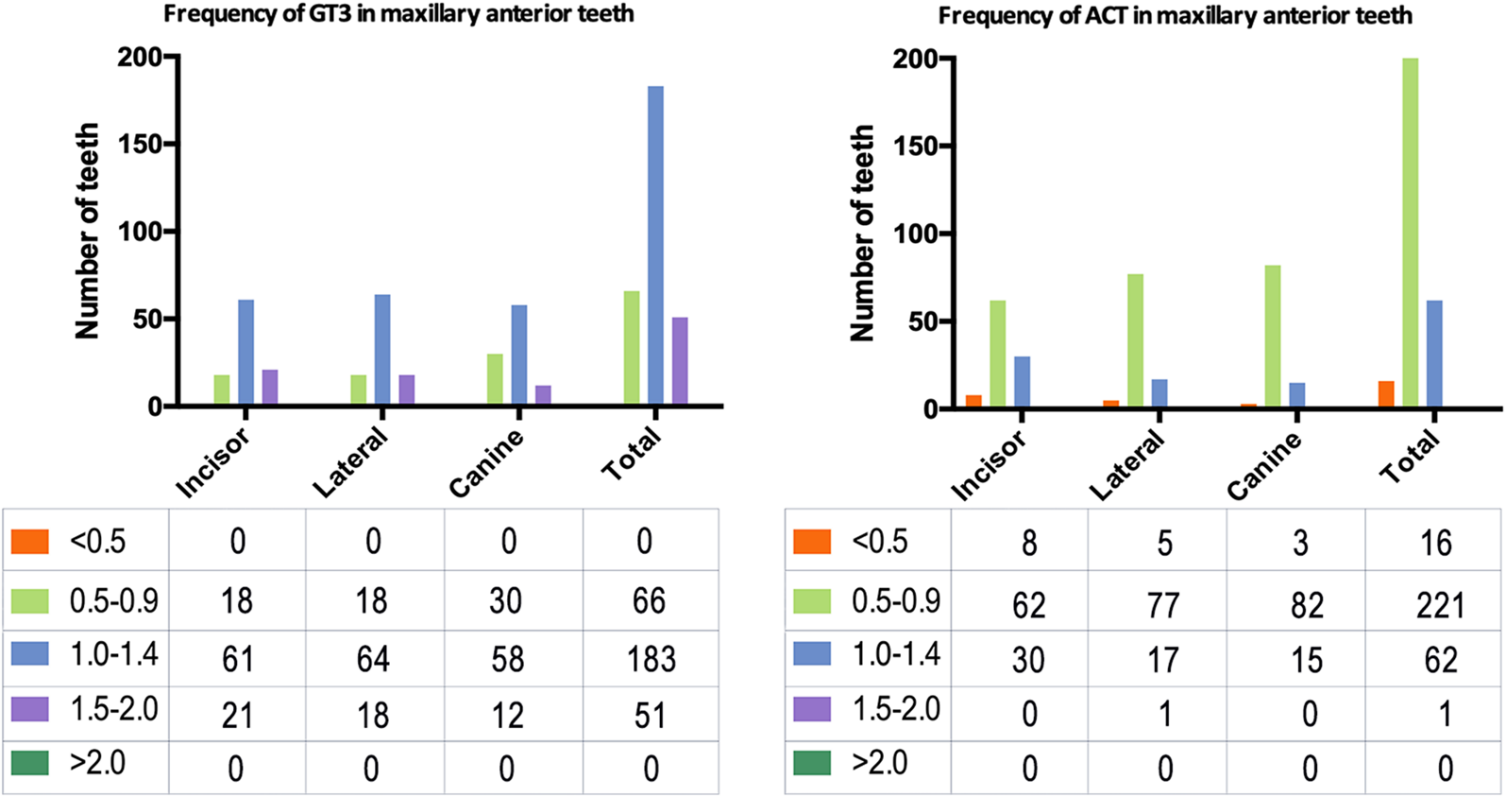
Frequency distribution of GT3 and ACT at maxillary anterior teeth. Tables (below each graph) show the number of teeth. Legends indicate different thickness ranges (from 0.5 to 2 mm)

Figure 2 shows the frequency distributions of labial GT3 and ACT measurements. For maxillary anterior teeth, a total of 66 teeth (22.0%) exhibited a GT3 of < 1.0 mm, 183 teeth (61.0%) exhibited a GT3 of 1.0–1.4 mm while 51 teeth (17.0%) exhibited a GT3 of 1.5–2.0 mm. No sites exhibited a GT3 of 2.0 mm or more. Furthermore, 16 teeth (5.3%) exhibited an ACT of < 0.5 mm, 221 teeth (73.7%) exhibited a thickness of 0.5–0.9 mm, 62 teeth (21.0%) exhibited a thickness of 1.0–1.4 mm while 1 tooth had an ACT of 1.5–2.0 mm. None of the teeth had an ACT of 2 mm or more.

## Discussion

Accurate measurements of soft and hard tissue thickness is important for establishing oral therapeutic outcomes, especially in aesthetically critical areas. In this study, 15-MHz B-mode US was used to detect labial periodontal structures and measure GT as well as ACT of the maxillary anterior teeth. Compared to the females, the males had a thicker labial GT3. In addition, the labial GT3 of canines were thinner than those of the central and lateral incisors. A correlation between labial GT3 and ACT revealed a moderately positive association. These results are consistent with a recent study that reported a moderately positive relationship between buccal bone and soft tissue thickness of the maxillary premolars [27]. Our study is the first to establish the use of 15-MHz B-mode US for simultaneous measurements of GT3, ACT and in the assessment of the correlation between the two parameters in the maxillary anterior teeth. This approach is non-invasive and does not involve radiation.

The thickness of periodontal tissues in maxillary anterior regions have been frequently analyzed in critical cases. In this study, we enrolled periodontally healthy young participants. This is because advancing age may influence GT [28]. The average GT3 of maxillary anterior teeth in the study sample group was 1.05 mm (canine, female)–1.31 mm (central incisor, male) which was comparable with a previous study reporting a maxilla GT of 0.89 mm (canines)–1.28 mm (central incisor) in humans using the A-mode US method [29]. A mode US probe can only obtain the GT value. Therefore, we used a 15-MHz B-mode probe to successfully calculate GT and ACT from intuitive anatomical images. Our results showed that the average GT3 in maxillary canines was thinner than those of the central and lateral incisors. This outcome may be due to prominence of a canine root and the mucosa was thinnest over the prominence hard tissue as previously reported [30].

Assessment of ACT is important for monitoring periodontal disease activity [3, 31]. In this study, the average ACT was 0.79, 0.76 and 0.73 mm for maxillary central incisor, lateral incisor and canine, respectively, which were generally thinner than the respective 0.89, 0.90 and 0.93 mm previously reported using the CBCT method at 1 mm below the ACT [32]. The differences could be attributed to the varying techniques and selection of measurement sites.

The females exhibited significantly thinner labial GT3 in the maxillary anterior teeth compared to males. The relationship between gender and GT has not been established. Some studies have documented that gender influences GT [22, 28, 33], while others have reported there being no significant differences between males and females with regard to maxillary masticatory mucosa [9, 34]. These differences could be attributed to varying tooth positions, different measuring sites, sample sizes as well as other confounding factors that influence GT such as racial and genetic factors [7].

In the current study, we chose 3 mm apical from the gingival margin as the measurement point, which is very close to the alveolar crest. The relationship between GT3 and ACT was then explored, following calculation of a mean value for ACT per tooth. Results revealed a moderate positive correlation between GT and ACT, which was consistent with some [35, 36] and inconsistent with other [37, 38] studies. Using CBCT scans, Fu et al. [35] reported that labial GT was moderately associated with underlying bone thickness from 22 cadavers and Amid et al.[36] found that the mean bone thickness was greater in patients with thick gingiva compared to those with thin gingiva. However, La Rocca et al. [37] and Stein et al. [38], demonstrated a negative correlation between labial bone thickness and GT as measured based on CBCT methods. These contradictory results indicate the morphologic variations of the periodontal tissue as previously suggested by Maynard and Wilson [39]. It is, therefore, necessary for clinicians to evaluate these parameters before treatment in order to avoid risks and select appropriate procedures.

## Conclusion

The 15-MHz B-mode US can be used to assess the labial periodontal tissue structure of anterior teeth. To the best of our knowledge, this is the first report regarding the use of 15-MHz B mode US to measure GT as well as ACT, and analyze their correlations in human anterior teeth. We found that GT3 was greater in men than in women. In addition, it was thinnest in maxillary anterior teeth canines. GT3 exhibited a moderate positive correlation with ACT. More studies are required to provide information for the appropriate assessment and predictable management of implant, periodontal and orthodontic patients in clinical practice.

## Data Availability

All necessary data are presented within the manuscript. All other materials and data are available upon request. For any more details regarding the data of this research please contact the corresponded author.

## Abbreviations

GT: Gingival thickness
GT3: 3 Mm apical to the gingival margin
ACT: Alveolar crest thickness
US: Ultrasonic
CBCT: Cone-beam computed tomography
